# Supplementary material for: Cell-fate conversion of intestinal cells in adult Drosophila midgut by depleting a single transcription factor
Source: Nat Commun. 2024 Mar 26;15:2656. doi: 10.1038/s41467-024-46956-8 (PMC10966032; doi:10.1038/s41467-024-46956-8)
Supplement: Supplementary file 1 — Supplementary Information [file 41467_2024_46956_MOESM1_ESM.pdf]

Supplementary Information

# Cell-fate conversion of intestinal cells in adult *Drosophila* midgut by depleting a single transcription factor

Xingting Guo[1, 2], Chenhui Wang[1, 3 *], Yongchao Zhang[1, 2], Ruxue Wei[1], Rongwen Xi[1, 2 *]

[1] National Institute of Biological Sciences, No. 7 Science Park Road, Zhongguancun Life Science Park, Beijing 102206, China

[2] Tsinghua Institute of Multidisciplinary Biomedical Research, Tsinghua University, Beijing, 102206, China.

[3] School of Life Science and Technology, ShanghaiTech University, Shanghai, 201210, China.

* Correspondence: wangchh5@shanghaitech.edu.cn or xirongwen@nibs.ac.cn

Supplementary Figures

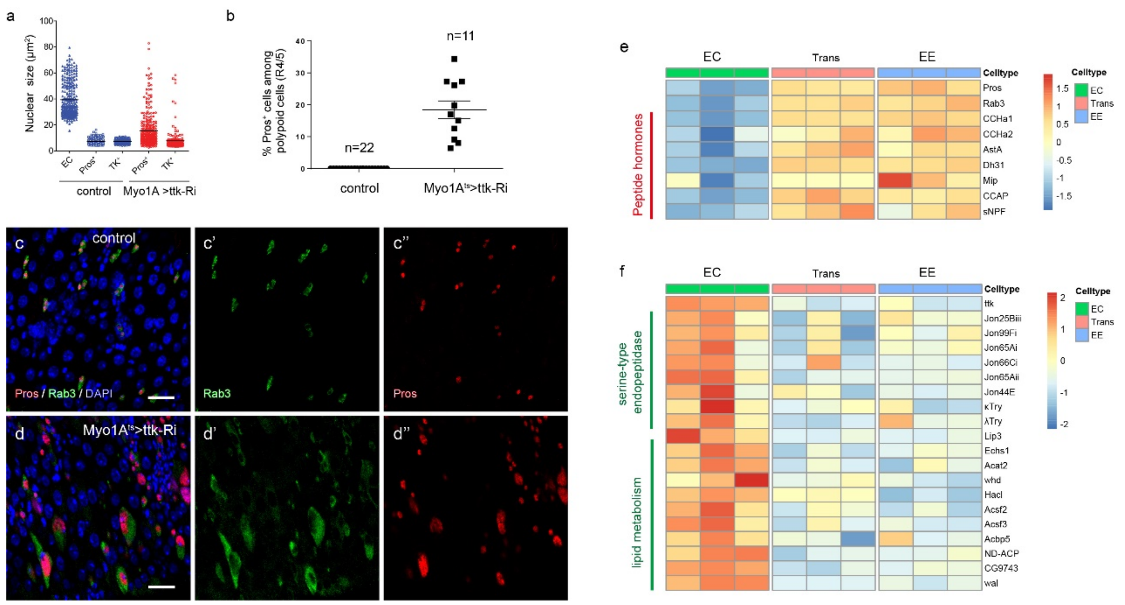

**Supplementary Figure 1. Activation of EE identity genes and suppression of EC function related genes in Ttk-depleted ECs. Related to Figures 1 and 2.**

(a) Ploidy analysis of Pros$^+$ or Tk$^+$ cells in normal and Myo1A$^{ts}$>ttk-RNAi guts. In normal guts, Pros and Tk are expressed only in diploid cells. In Myo1A$^{ts}$>ttk-RNAi guts, Pros or Tk expression is found in a subset of polyploid cells. Cells with nuclear size>20 $\mu m^2$ were defined as polyploidy.

(b) Quantification of Pros$^+$ cells in normal and Ttk-depleted ECs. Mean ± SEM, "n" indicate the number of guts used for quantification; source data are provided as a Source Data file.

(c-d) Rab3 is only expressed in EEs in normal gut (c). Its expression is found in some ECs following Ttk depletion (d).

(e) Heatmap showing the mRNA level of multiple EC identity genes is significantly upregulated in Ttk-depleted ECs, including the pan-EE TF Prospero, the secretory process component Rab3, and multiple peptide hormones.

(f) Heatmap showing the mRNA level of multiple EC function related genes is significantly downregulated in Ttk-depleted ECs, including genes encoding serine-type endopeptidase and proteins involved in lipid metabolic processes.

Scale bars, 20 µm

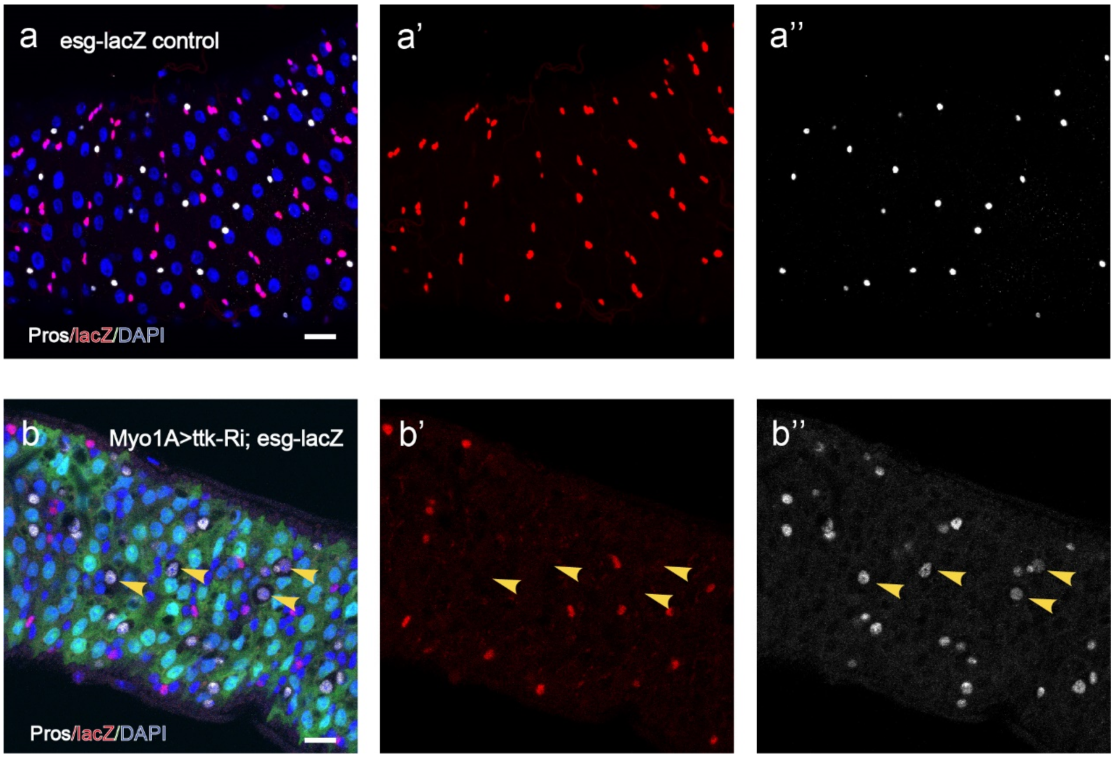


**Supplementary Figure 2. Ttk-depletion induces direct EC to EE transdifferentiation. Related to Figure 1.** No progenitor marker esg-lacZ could be observed in Ttk-depleted polyploid cells (b).

Scale bars, 20 μm

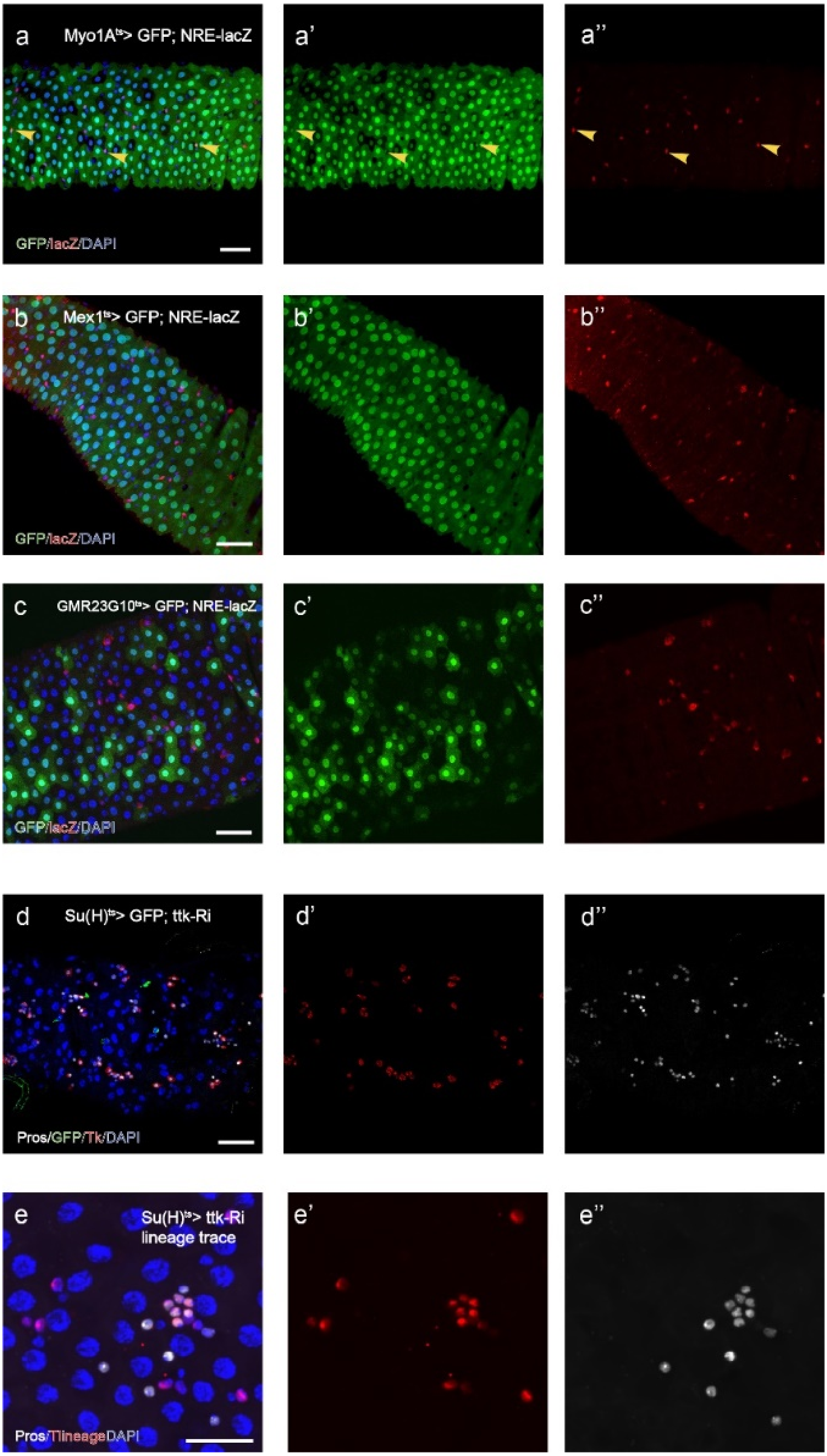
a
Myo1A^ts> GFP; NRE-lacZ
GFP/lacZ/DAPI
a'
a''
b
Mex1^ts> GFP; NRE-lacZ
GFP/lacZ/DAPI
b'
b''
c
GMR23G10^ts> GFP; NRE-lacZ
GFP/lacZ/DAPI
c'
c''
d
Su(H)^ts> GFP; ttk-Ri
Pros/GFP/Tk/DAPI
d'
d''
e
Su(H)^ts> ttk-Ri
lineage trace
Pros/TlineageDAPI
e'
e''

**Supplementary Figure 3. The transdifferentiated Polyploid Pros+ cells are derived from ECs, not EBs. Related to Figures 1 and 2.**

(a) The EB maker NRE-lacZ is expressed in part of Myo1A-GFP$^+$ cells (yellow arrowheads, with small nuclear size).

(b-c) The EB maker NRE-lacZ is not expressed in GFP$^+$ cells driven by Mex1-Ga4 (b) or GMR23G10-Gal4 (c), two additional EC-specific drivers.

(d) Knocking down Ttk in EC-committed EBs leads to accumulation of diploid EE cell clusters, but no polyploid Pros$^+$ cells could be observed.

(e) Lineage tracing analysis using Su(H)-GBE-Gal4 shows that EBs with Ttk depletion give rise to diploid but not polyploid Pros$^+$ cells.

Scale bars, 50 μm.

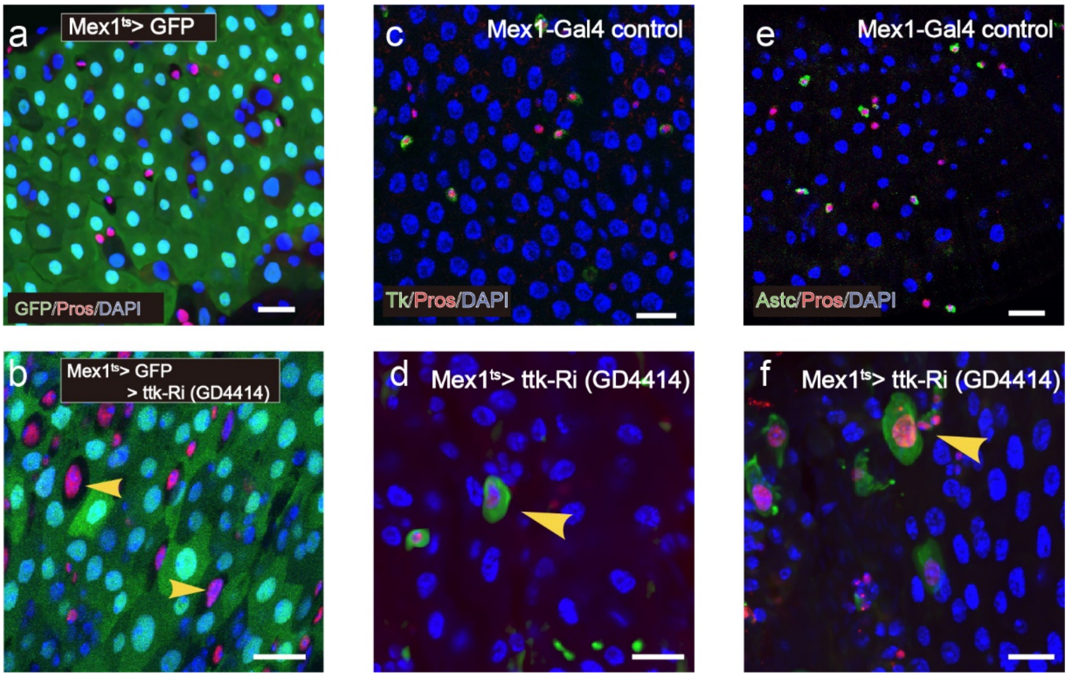


**Supplementary Figure 4. Knocking down Ttk using Mex1-Gal4 leads to EC-to-EE transdifferentiation. Related to Figures 1 and 2.**

(a-b) Knocking down Ttk in ECs using Mex1-Gal4 induces the appearance of Pros$^+$ polyploid cells, and GFP expression in these transdifferentiated cells is suppressed (b).

(c-f) Tk or AstC expression is observed in the transdifferentiated cells (d, f, yellow arrowhead);

Scale bars, 20 μm.

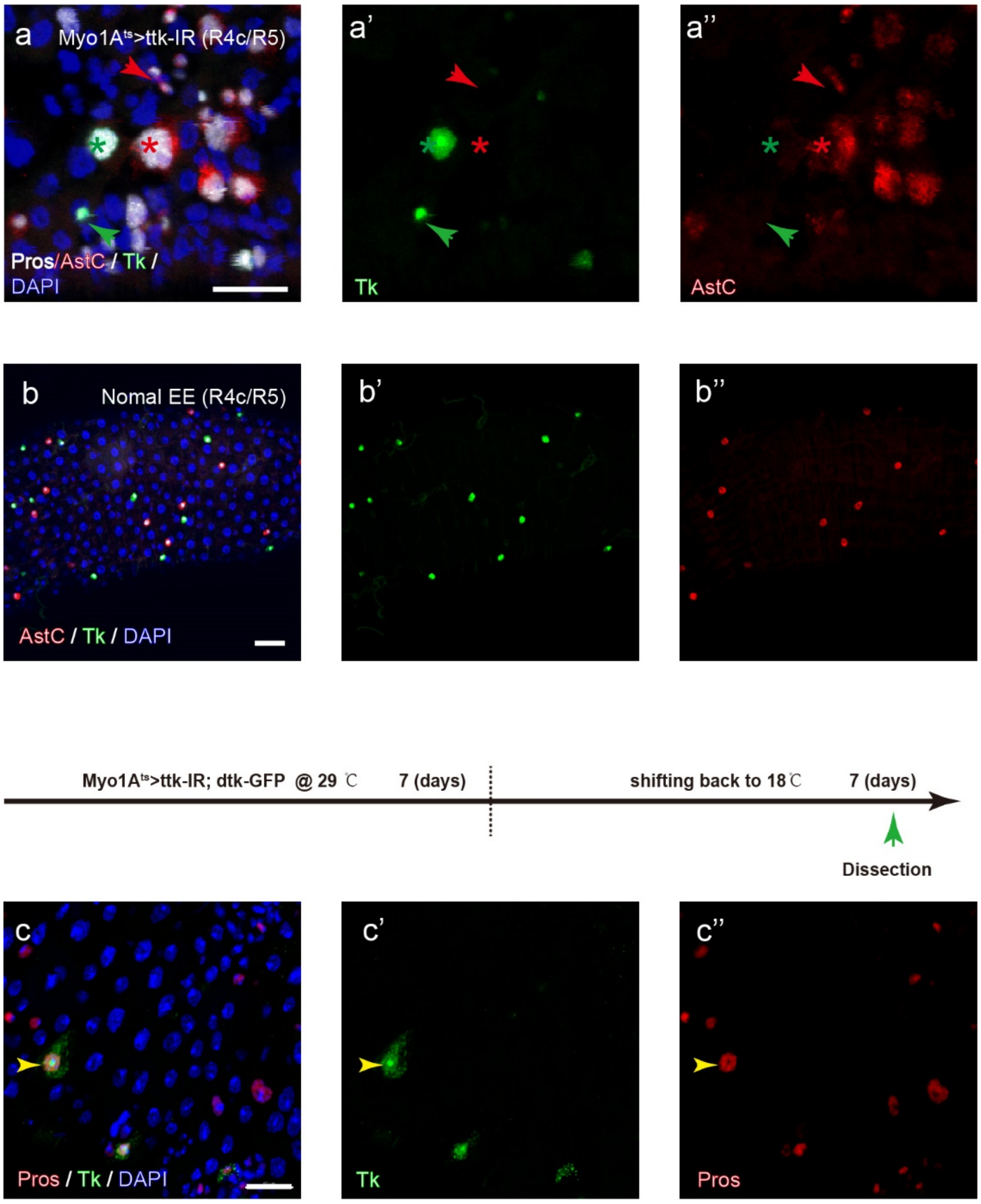


**Supplementary Figure 5. The transdifferentiated cells exhibit subtype diversity similar to normal EEs. Related to Figure 2.**

(a-b) Tk or AstC expression can be observed in the transdifferentiated cells, and similar to that in normal EEs (b), the expression of Tk (a, green star) and AstC (a, red star) in the transdifferentiated cells is also mutually exclusive.

(c) The identity of the transdifferentiated cells persists after shifting Myo1A>ttk-RNAi flies back to 18℃ culture for 7 days (yellow arrowheads);

Scale bars, 20 μm.

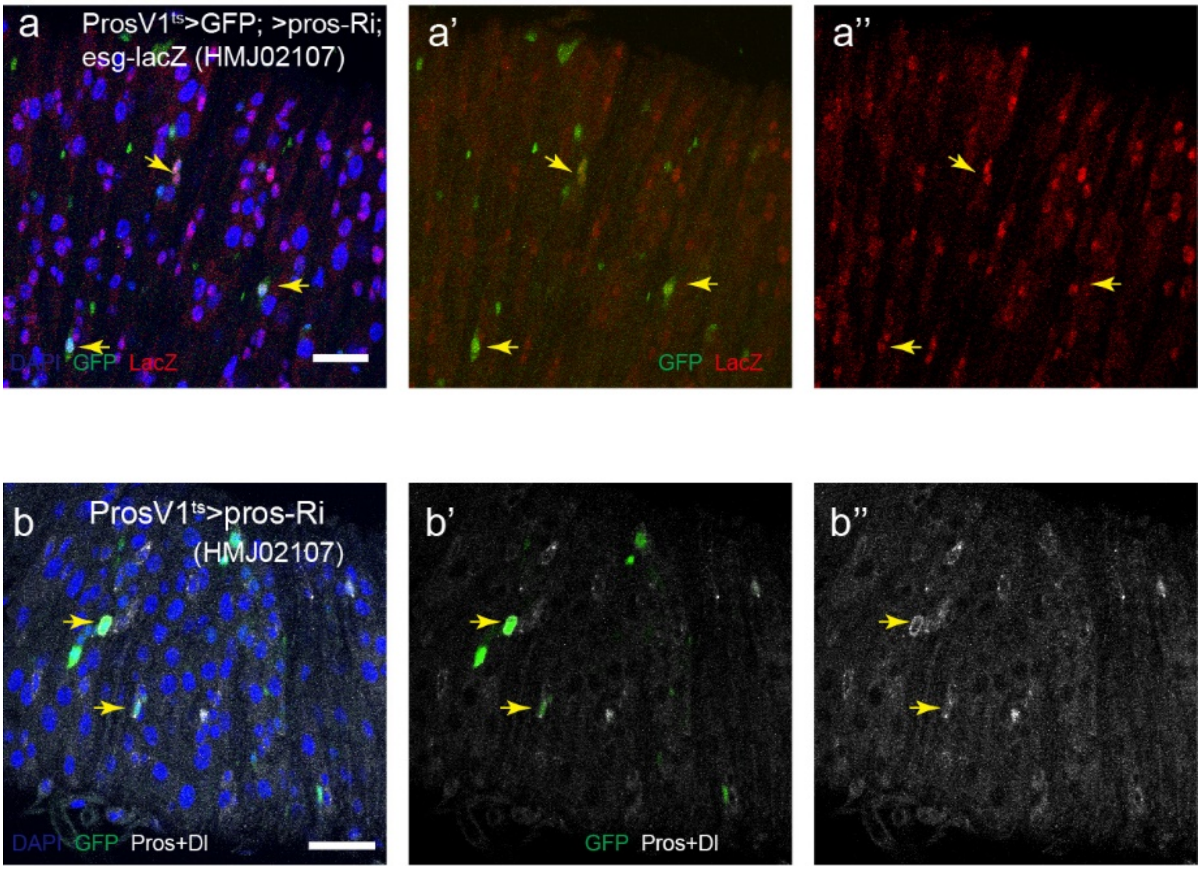


**Supplementary Figure 6. Knocking down Pros in EEs with an independent RNAi line (TRiP.HMJ02107) leads to EE-to-ISC transdifferentiation. Related to Figure 4.**

Note the turn on of progenitor cell marker esg-lacZ (a, yellow arrows) and Dl (b, yellow arrows) in prosV1>GFP$^{+}$ cells.

Scale bars, 50 μm.

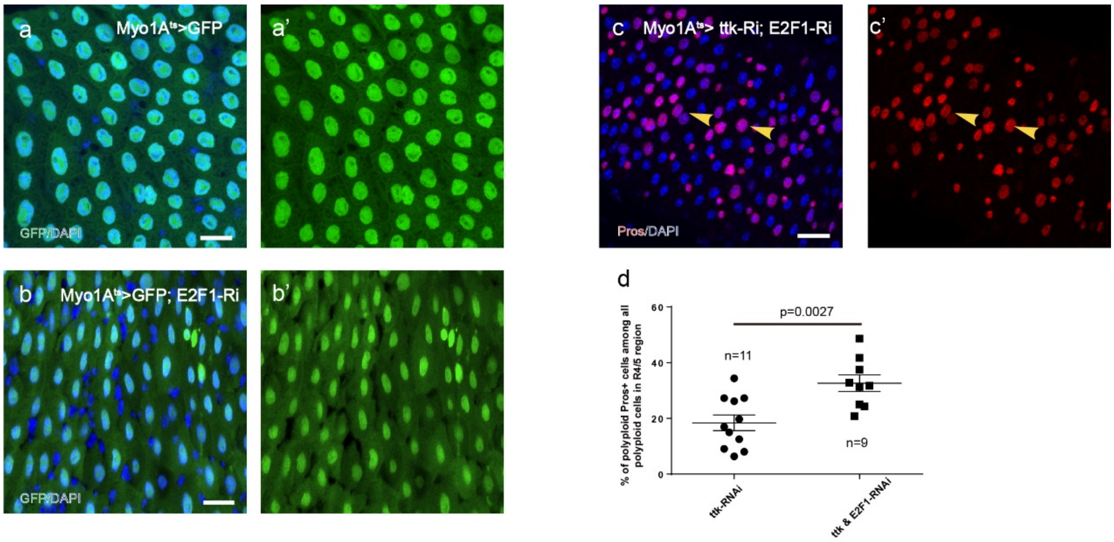


**Supplementary Figure 7. Polyploidy might be a barrier to cell transdifferentiation. Related to Figure 6.**

(a-b) Knocking down E2F1 using Myo1A-Gal4 leads to decreased nuclear sizes in ECs.

(c-d) Knocking down both Ttk and E2F1 using Myo1A-GAL4 leads to significant increase of Tk$^+$ polyploid cells. **p<0.01 (two-tailed Student's t test). "n" indicate the number of guts used for quantification; source data are provided as a Source Data file.

Scale bars, 20 μm.

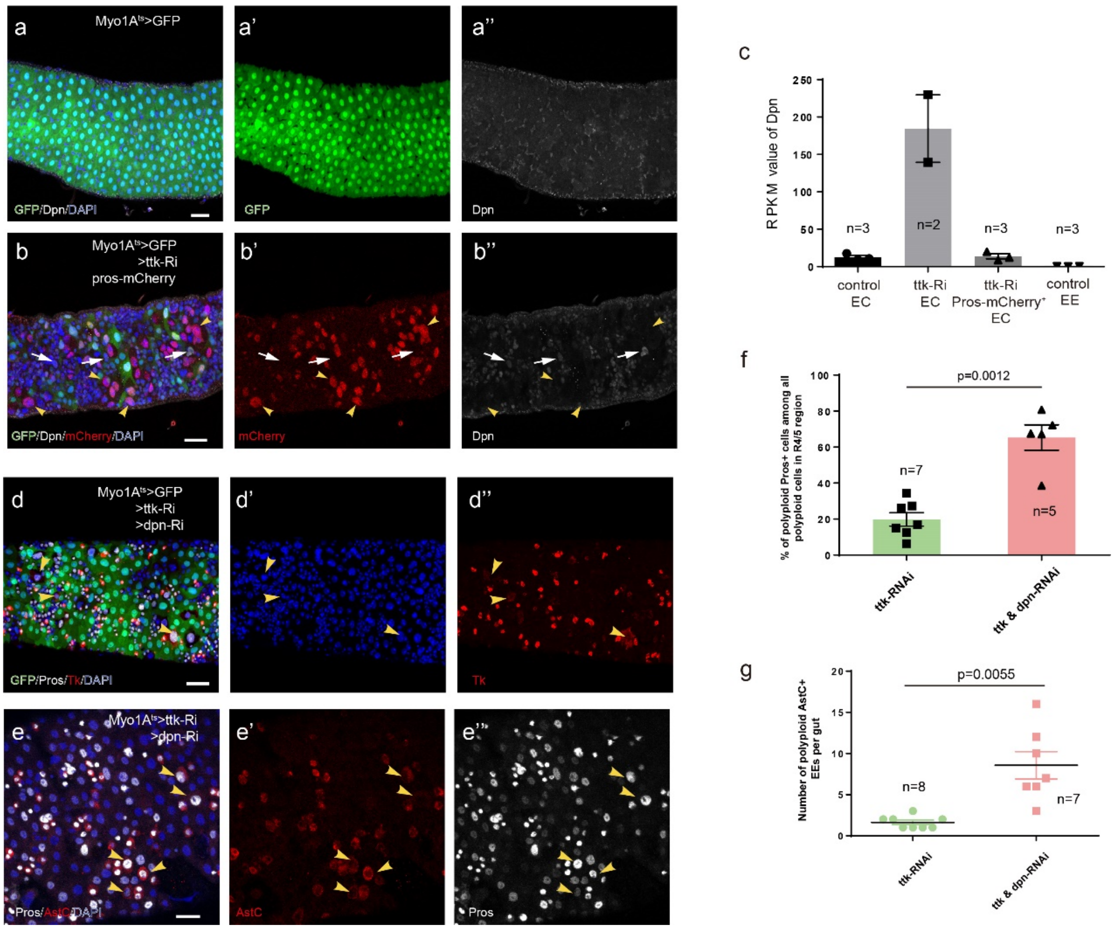


**Supplementary Figure 8. Lineage confliction as a barrier to the EC-to-EE transdifferentiation. Related to Figure 6.**

(a-b) Dpn is not expressed in normal guts (a); Depleting Ttk in ECs causes Dpn derepression (b, white arrow), and co-staining with Pros (b, yellow arrowheads) reveals an inverse correlation between the expression of Dpn and Pros.

(c) The transcription levels of *dpn* in normal EC and EE, as well as in Ttk-depleted ECs without or with Pros activation. Error bars represent Mean ± SEM, "n" indicate the replicates of RNA-seq; source data are provided as a Source Data file.

(d-e) Knocking down both *ttk* and *dpn* using Myo1A1-GAL4 leads to significant increase of $Tk^+$ (d, yellow arrowhead) and $AstC^+$(e, yellow arrowhead) polyploid cells;

(f-g) Quantification of $Pros^+$ (f) and $AstC^+$ (g) polyploid cells upon *ttk*-knock down alone or *ttk* and *dpn* together. Error bars represent Mean ± SEM, ***p<0.001 (two-tailed Student's t test), "n" indicate the number of guts used for quantification; source data are provided as a Source Data file.

Scale bars, 20 μm.

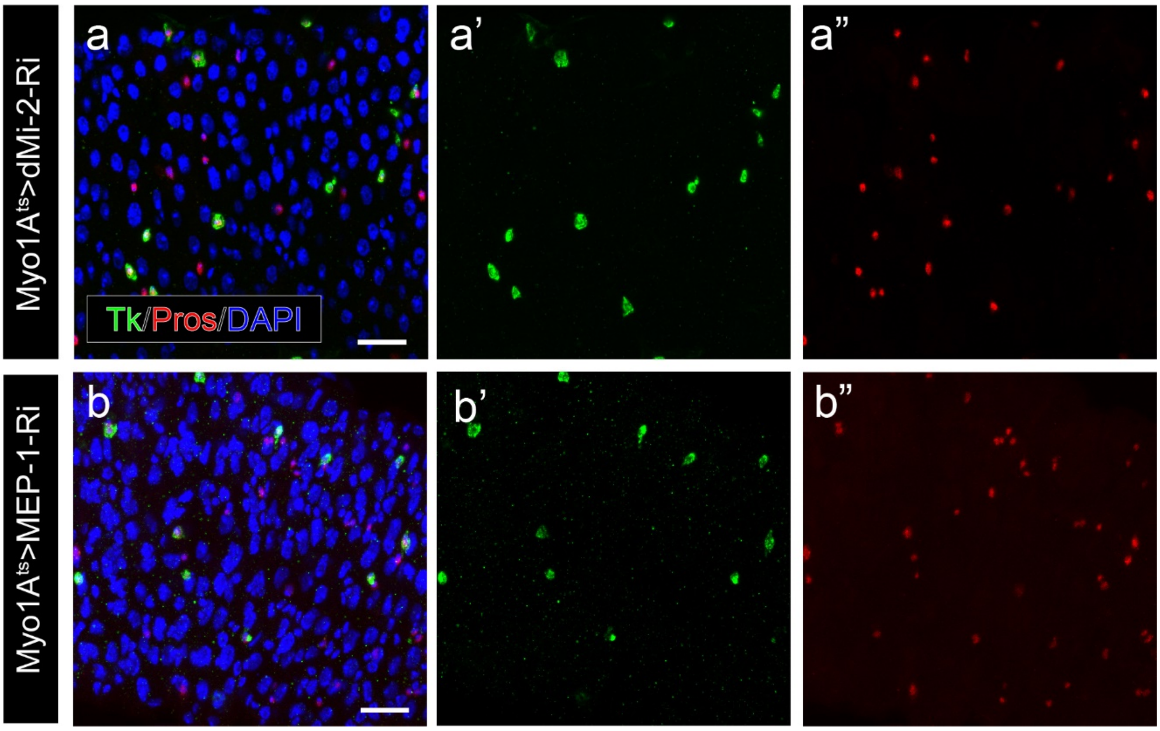


**Supplementary Figure 9. Depletion of Mi-2 or MEP1 in ECs does not cause any obvious phenotype. Related to Figure 7.**

(a-b) Depletion of either dMi-2 (a) or MEP1 (b) using Myo1A-GAL4 does not induce the expression of Pros or Tk expression in ECs.

Scale bars, 20 μm.

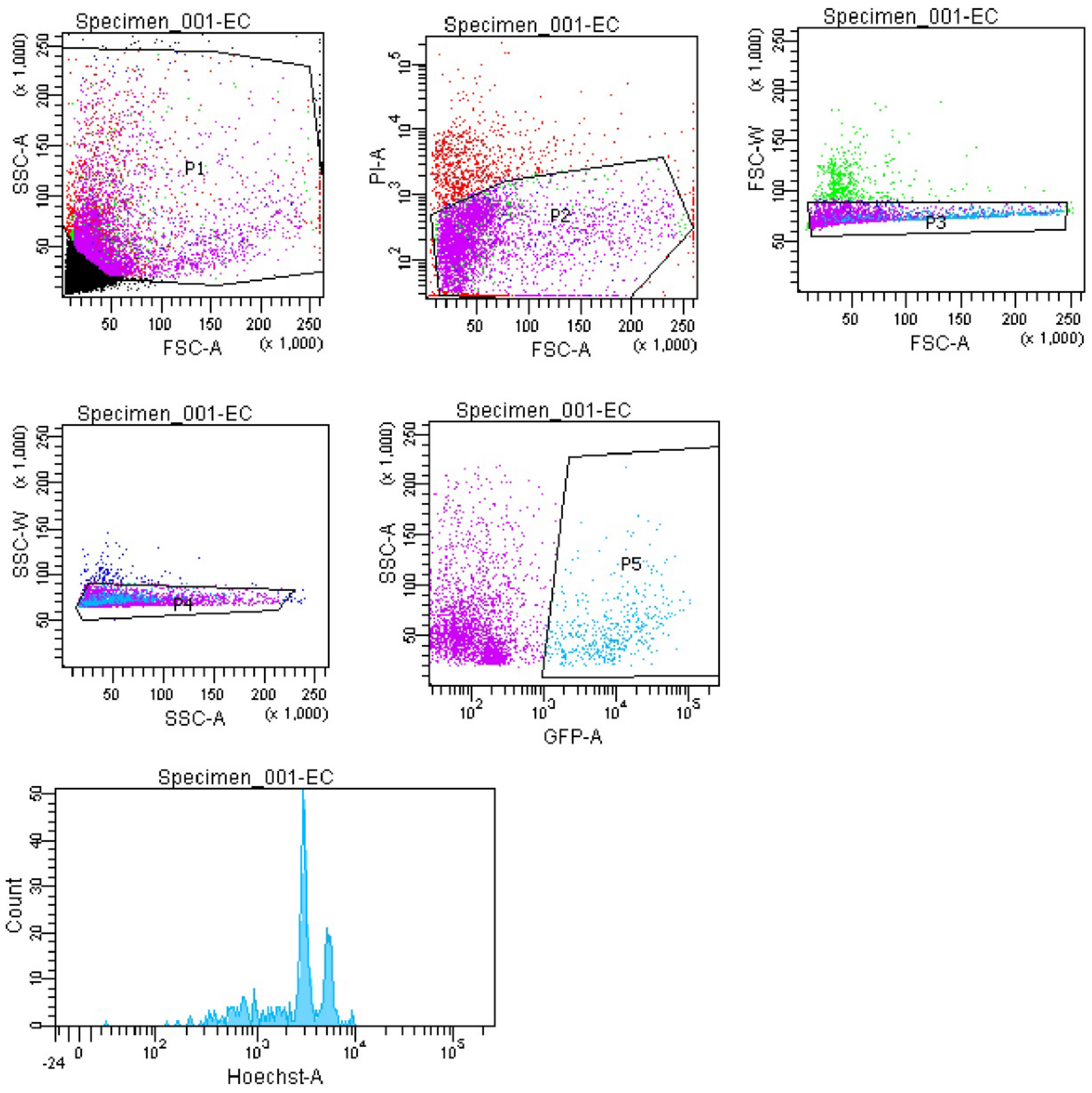


Tube: EC

| Population | #Events | %Parent | %Total |
|---|---|---|---|
| All Events | 100,000 | #### | 100.0 |
| P1 | 5,947 | 5.9 | 5.9 |
| P2 | 4,138 | 69.6 | 4.1 |
| P3 | 3,645 | 88.1 | 3.6 |
| P4 | 3,516 | 96.5 | 3.5 |
| P5 | 476 | 13.5 | 0.5 |

**Supplementary Figure 10. FACS sequential gating/sorting strategies. Related to Figures 2, 3, and 5.**

$GFP^+$ cells in P5 are collected and used for ATAC- and RNA-sequencing analysis.
