# Supplementary material for: Cell-fate conversion of intestinal cells in adult Drosophila midgut by depleting a single transcription factor
Source: Nat Commun. 2024 Mar 26;15:2656. doi: 10.1038/s41467-024-46956-8 (PMC10966032; doi:10.1038/s41467-024-46956-8)
Supplement: Supplementary file 3 — Description of Additional Supplementary Files [file 41467_2024_46956_MOESM3_ESM.pdf]

Description of Additional Supplementary Files

File Name: Supplementary Data 1
Description: Differently expressed genes among EC, EE and ttk-depletion induced transformed EEs. Related to figure 2g.

File Name: Supplementary Data 2
Description: Transcriptome of normal EE, EC, and u-Pros or ttk-IR&pros-IR depleted ECs. Related to Figure 3i.

File Name: Supplementary Data 3
Description: Gene list of significantly up-regulated genes in pros depleted EEs. Related to Figure 4a-c.

File Name: Supplementary Data 4
Description: List of top 250 progenitor identity genes. Related to figure 4b.

File Name: Supplementary Data 5
Description: List of epigenetic regulators and RNAi screen for additional barriers. Related to Figure 7.
