# Supplementary material for: Cell-fate conversion of intestinal cells in adult Drosophila midgut by depleting a single transcription factor
Source: Nat Commun. 2024 Mar 26;15:2656. doi: 10.1038/s41467-024-46956-8 (PMC10966032; doi:10.1038/s41467-024-46956-8)
Supplement: Supplementary file 9 — Reporting Summary [file 41467_2024_46956_MOESM9_ESM.pdf]

nature portfolio

Corresponding author(s): Rongwen Xi

Last updated by author(s): Mar 5, 2024

Reporting Summary

Nature Portfolio wishes to improve the reproducibility of the work that we publish. This form provides structure for consistency and transparency in reporting. For further information on Nature Portfolio policies, see our Editorial Policies and the Editorial Policy Checklist.

Statistics

For all statistical analyses, confirm that the following items are present in the figure legend, table legend, main text, or Methods section.

n/a	Confirmed	
☐	☒	The exact sample size (n) for each experimental group/condition, given as a discrete number and unit of measurement
☐	☒	A statement on whether measurements were taken from distinct samples or whether the same sample was measured repeatedly
☐	☒	The statistical test(s) used AND whether they are one- or two-sided<br>*Only common tests should be described solely by name; describe more complex techniques in the Methods section.*
☒	☐	A description of all covariates tested
☐	☒	A description of any assumptions or corrections, such as tests of normality and adjustment for multiple comparisons
☐	☒	A full description of the statistical parameters including central tendency (e.g. means) or other basic estimates (e.g. regression coefficient) AND variation (e.g. standard deviation) or associated estimates of uncertainty (e.g. confidence intervals)
☐	☒	For null hypothesis testing, the test statistic (e.g. F, t, r) with confidence intervals, effect sizes, degrees of freedom and P value noted<br>*Give P values as exact values whenever suitable.*
☒	☐	For Bayesian analysis, information on the choice of priors and Markov chain Monte Carlo settings
☒	☐	For hierarchical and complex designs, identification of the appropriate level for tests and full reporting of outcomes
☒	☐	Estimates of effect sizes (e.g. Cohen's d, Pearson's r), indicating how they were calculated

Our web collection on statistics for biologists contains articles on many of the points above.

Software and code

Policy information about availability of computer code

Data collection	NIKON NIS Elements (version 5.21.00); BD FACSDiva (version 6.1.3)
Data analysis	Bulk RNA-sequencing: Raw reads were mapped to D. melanogaster genome (BDGP6) and counts assigned to protein-coding genes were calculated using featureCounts (v1.6.3). DESeq2 was then used to identify significantly differently expressed genes using the following parameters: Padj<0.01, and the absolute value of log2 FC>0.5. GO analysis for differently expressed genes was performed using DAVID 42, and the R package “pheatmap” was used for generating heatmaps.<br>ATAC-seq analysis: Raw sequencing reads were mapped to the D. melanogaster genome (BDGP6) using bowtie2 (version 2.2.4). To generate bigWig (bw) files, the deepTools bamCoverage function was employed with BPM normalization. Finally, peaks were called using MACS3 to identify regions of enriched signal.<br>The GSEA analysis was performed done using the R package “clusterprofiler”;<br>ImageJ software (version 1.48v) was utilized for cell number counting.<br>GraphPad Prism 6 software (GraphPad Software Inc.) was used to calculate p-values by unpaired student’s t-test or anova test.

For manuscripts utilizing custom algorithms or software that are central to the research but not yet described in published literature, software must be made available to editors and reviewers. We strongly encourage code deposition in a community repository (e.g. GitHub). See the Nature Portfolio guidelines for submitting code & software for further information.

Data

Policy information about availability of data

All manuscripts must include a data availability statement. This statement should provide the following information, where applicable:

- Accession codes, unique identifiers, or web links for publicly available datasets
- A description of any restrictions on data availability
- For clinical datasets or third party data, please ensure that the statement adheres to our policy

The raw and processed datasets, including RNA-seq data and ATAC-seq results generated in this study, have been made available in the supplementary material or deposited in the GEO database under the accession code GSE235505 (https://www. ncbi.nlm.nih.gov/geo/query/acc.cgi?acc=GSE235505). The genome datasets used in this study was BDGP6 for RNA-seq and ATAC-seq analysis. Additionally, three RNA-seq datasets previously reported by our lab are accessible in the GEO database under the following accession codes: GSE130943 (RNA-seq data of esg+ cell; https://www. ncbi.nlm.nih.gov/geo/query/acc.cgi?acc=GSE130943 42), GSE130305 (RNA-seq data of ECs; https://www.ncbi.nlm.nih.gov/geo/query/acc.cgi?acc= GSE130305 26), and GSE211632 (RNA-seq for control EE and Pros-depleted EE; https://www.ncbi.nlm.nih. gov/geo/query/acc.cgi?acc=GSE211632 15). Furthermore, several public ATAC-seq datasets related to multiple tissues were utilized in this study: GSE157776 (intestinal stem cells; https://www.ncbi.nlm.nih.gov/geo/query/acc.cgi? acc=GSE157776 30), GSE59078 (Drosophila eye-antennal discs from wandering third instar larvae; https://www.ncbi.nlm.nih.gov/geo/query/acc.cgi?acc=GSE59078 31), SRX9186391-SRX9186393 (2-day-old Drosophila whole testis; https://www.ncbi.nlm. nih.gov/bioproject/PRJNA665509 63), GSE81434 (OSC, ovarian somatic cells; https://www. ncbi.nlm.nih.gov/geo/query/acc.cgi?acc=GSE81434 32), and GSE154645 (FACS sorted GFP+ neurons from larval brain, elav>Dcr-2, mCD8::GFP; https://www. ncbi.nlm.nih.gov/geo/query/acc.cgi?acc=GSE154645;) 33. Source data are provided with this paper.

Human research participants

Policy information about studies involving human research participants and Sex and Gender in Research.

Reporting on sex and gender	N/A
Population characteristics	N/A
Recruitment	N/A
Ethics oversight	N/A

Note that full information on the approval of the study protocol must also be provided in the manuscript.

Field-specific reporting

Please select the one below that is the best fit for your research. If you are not sure, read the appropriate sections before making your selection.

☒ Life sciences ☐ Behavioural & social sciences ☐ Ecological, evolutionary & environmental sciences

For a reference copy of the document with all sections, see nature.com/documents/nr-reporting-summary-flat.pdf

Life sciences study design

All studies must disclose on these points even when the disclosure is negative.

Sample size	Pre-determining sample sizes is not necessary, as the number of Drosophila is not limited. Sample sizes were determined based on variations between different genotypes and treatments. At least 2-3 replicates were carried out for each experiment.
Data exclusions	No data were excluded from these analysis.
Replication	All experiments were reproduced for at least 2-3 times and representative results were shown in the manuscript. For key results, independent RNAi lines targeting to the same genes were also used to verify these findings.
Randomization	The flies with same genotypes were collected together and been randomly allocated into different groups or treatments.
Blinding	Blinding was performed in all quantifications. Other experiments were not blinded to the investigators, as the genotypes of the fly strains should be visible to the investigators to carry out appropriate treatment, and staining with appropriate antibody .

Reporting for specific materials, systems and methods

We require information from authors about some types of materials, experimental systems and methods used in many studies. Here, indicate whether each material, system or method listed is relevant to your study. If you are not sure if a list item applies to your research, read the appropriate section before selecting a response.

Materials & experimental systems

n/a	Involved in the study
☐	☒ Antibodies
☒	☐ Eukaryotic cell lines
☒	☐ Palaeontology and archaeology
☐	☒ Animals and other organisms
☒	☐ Clinical data
☒	☐ Dual use research of concern

Methods

n/a	Involved in the study
☒	☐ ChIP-seq
☐	☒ Flow cytometry
☒	☐ MRI-based neuroimaging

Antibodies

Antibodies used	Primary antibodies used in this study were as follows: mouse anti-Pros (DSHB #MR1A; 1:300); mouse monoclonal anti-β-galactosidase (DSHB,# 40-1a; 1:30); mouse anti-Dl (DSHB Cat#C594.9B; RRID:AB_528194; 1:300); rabbit anti-AstC (lab generated antibody (RRID: AB_2753141) and gift from Dr. Dick Nassel; 1:300); rabbit anti-Tk (lab generated antibody (RRID: AB_2569591) and gift from Dr. Jan-Adrianus Veenstra; 1:300); rabbit polyclonal anti-β-galactosidase (Cappel, 0855976; 1: 3000); Rabbit anti-pH3 (CST Cat# 9701; RRID:AB_331535; 1:500); Rabbit anti-Pdm1 (lab generated antibody and gift from Dr. Xiaohang Yang; 1:200); Rabbit anti-Dpn (lab generated antibody and gift from Dr.Yuh-NungJan, RRID:AB_2567048;1:250); Rabbit anti-Sox100b (lab generated antibody in our lab; 1:500); Rabbit anti-Ttk69 (lab generated antibody in our lab; 1:200); Rabbit anti-Sox21a (lab generated antibody in our lab; 1:100).<br>Secondary antibodies used in this study include Alexa Fluor 488-, 568- or Cy5-conjugated goat anti-rabbit, anti-mouse IgGs (Molecular Probes, A11034-A11036, A10524; 1:300). For nuclei staining, DAPI (Sigma-Aldrich, 1μg/ml) was used.
Validation	All antibodies used in this study have been validated by previous work specifically in Drosophila midgut. mouse anti-Pros (DSHB #MR1A; 1:300); rabbit anti-AstC (gift from Dr. Dick Nassel); rabbit anti-Tk (gift from Dr. Jan-Adrianus Veenstra) (PMID: 31851941) rabbit polyclonal anti-β-galactosidase (Cappel, 0855976; 1: 6000); mouse monoclonal anti-β-galactosidase (DSHB,# 40-1a; 8:300) (PMID: 32460025); Rabbit anti-Dpn (lab generated antibody and gift from Dr.Yuh-NungJan, RRID:AB_2567048;1:250) (PMID: 32049006); Rabbit anti-Sox21a (lab generated antibody in our lab) (PMID: 27187149)

Animals and other research organisms

Policy information about studies involving animals; ARRIVE guidelines recommended for reporting animal research, and Sex and Gender in Research

Laboratory animals	The study involves the use of adult female flies. Adult female flies with correct genotypes were collected and used for subsequent genetic manipulation for 7d in most experiments. Details for treatments were presented in the figures and the main text.
Wild animals	This study did not use wild animals
Reporting on sex	Adult female flies were used in this study.
Field-collected samples	This study did not use field-collected samples.
Ethics oversight	The use of Drosophila does not require ethical approval.

Note that full information on the approval of the study protocol must also be provided in the manuscript.

Flow Cytometry

Plots

Confirm that:

☒ The axis labels state the marker and fluorochrome used (e.g. CD4-FITC).

☒ The axis scales are clearly visible. Include numbers along axes only for bottom left plot of group (a 'group' is an analysis of identical markers).

☒ All plots are contour plots with outliers or pseudocolor plots.

☒ A numerical value for number of cells or percentage (with statistics) is provided.

Methodology

Sample preparation	100-150 guts for each sample were dissected in ice-cold DEPC-PBS within 2 hours, and were digested in 1mg/ml elastase solution (Sigma, cat. no. E0258) for 1 hour at room temperature with gentle shakes. The dissociated samples were then centrifuged at 500g for 10 min at 4 °C, and re-suspended in 500μl DEPC-PBS with 1mg/ml propidium iodide (PI, Invitrogen, #P3566).
Instrument	Cells were collected using a FACS Aria II sorter (BD Biosciences)

Software	BD FACSDiva (version 6.1.3)
Cell population abundance	Around 20000 PI- GFP+ cells were sorted for each sample.
Gating strategy	First side scatter (SSC-A, log) and forward scatter (FSC-A, log) are plotted to eliminate fragments in the cell suspension; second, PI-561nm and FSC-A are plotted to eliminate dead cells. Thirdly, SSC-A and SSC-W are plotted to exclude cell clusters, and finally FITC (GFP) and SSC-A are plotted to collect GFP+ cells.

☒ Tick this box to confirm that a figure exemplifying the gating strategy is provided in the Supplementary Information.
